# Historic recombination in a durum wheat breeding panel enables high-resolution mapping of Fusarium head blight resistance quantitative trait loci

**DOI:** 10.1038/s41598-020-64399-1

**Published:** 2020-05-05

**Authors:** Ehsan Sari, Ron E. Knox, Yuefeng Ruan, Maria Antonia Henriquez, Santosh Kumar, Andrew J. Burt, Richard D. Cuthbert, David J. Konkin, Sean Walkowiak, Heather L. Campbell, Asheesh K. Singh, Jay Ross, Prabhath Lokuruge, Emma Hsueh, Kerry Boyle, Christine Sidebottom, Janet Condie, Shawn Yates, Curtis J. Pozniak, Pierre R. Fobert

**Affiliations:** 1 Aquatic and Crop Resource Development Centre, National Research Council, Saskatoon, SK Canada; 2 Swift Current Research and Development Centre, Agriculture and Agri-Food Canada, Swift Current, SK Canada; 3 Morden Research and Development Centre, Agriculture and Agri-Food Canada, Morden, MB Canada; 4 Brandon Research and Development Centre, Agriculture and Agri-Food Canada, Brandon, MB Canada; 5 Ottawa Research and Development Centre, Agriculture and Agri-Food Canada, Ottawa, ON Canada; 6 Department of Plant Sciences, University of Saskatchewan, Saskatoon, SK Canada; 7 Department of Agronomy, Iowa State University, Ames, Iowa United States of America; 8 Aquatic and Crop Resource Development Centre, National Research Council, Ottawa, ON Canada; 9 Present Address: Department of Plant Sciences, University of Saskatchewan, Saskatoon, SK Canada; 10 Present Address: Canadian Grain Commission, Winnipeg, MB Canada

**Keywords:** Plant biotechnology, Plant breeding, Plant genetics, Plant sciences

## Abstract

The durum wheat line DT696 is a source of moderate Fusarium head blight (FHB) resistance. Previous analysis using a bi-parental population identified two FHB resistance quantitative trait loci (QTL) on chromosome 5A: 5A1 was co-located with a plant height QTL, and 5A2 with a major maturity QTL. A Genome-Wide Association Study (GWAS) of DT696 derivative lines from 72 crosses based on multi-environment FHB resistance, plant height, and maturity phenotypic data was conducted to improve the mapping resolution and further elucidate the genetic relationship of height and maturity with FHB resistance. The Global Tetraploid Wheat Collection (GTWC) was exploited to identify durum wheat lines with DT696 allele and additional recombination events. The 5A2 QTL was confirmed in the derivatives, suggesting the expression stability of the 5A2 QTL in various genetic backgrounds. The GWAS led to an improved mapping resolution rendering the 5A2 interval 10 Mbp shorter than the bi-parental QTL mapping interval. Haplotype analysis using SNPs within the 5A2 QTL applied to the GTWC identified novel haplotypes and recombination breakpoints, which could be exploited for further improvement of the mapping resolution. This study suggested that GWAS of derivative breeding lines is a credible strategy for improving mapping resolution.

## Introduction

Fusarium head blight (FHB) is a devastating disease of wheat causing severe reduction in grain yield and quality in warm and moist growing regions of the world. The causal pathogen *Fusarium graminearum* Schwabe produces mycotoxins that are harmful to human and animal health^1^. The contaminated grains are downgraded because they are unhealthy for human and animal consumption. Integrated FHB management provides the most effective control strategy and utilizes fungicide application, residue management, crop rotation, and growing cultivars with the highest available level of resistance. Growing cultivars with resistance is an efficient and cost-effective component of the strategy, consequently developing FHB resistant cultivars is one of the major objectives of wheat breeding programs where the disease is a problem. Only partial resistance has thus far been reported in bread wheat germplasm and durum wheat cultivars lack adequate resistance to FHB. The durum wheat line DT696 is a source of moderate FHB resistance adapted to the western Canadian Prairies^2^. Durum wheat cultivars derived from DT696 as a source of FHB resistance, such as Brigade, Transcend, and CDC Credence show improved FHB resistance relative to other elite durum wheat cultivars in production^3,4^. Using resistance available in adapted sources such as DT696 has the advantage of minimizing detrimental effects of linkage drag since DT696 is an advanced elite breeding line adapted to Canadian growing conditions.

High-density mapping of FHB resistance quantitative trait loci (QTL) using a large bi-parental population (number of lines 427) from a cross between lines DT707 and DT696 identified five resistance QTL on chromosomes 1B, 2B, 5A (two loci), and 7A^2^. The QTL on chromosome 5A (5A1 and 5A2) were consistently expressed over different field locations and years. The 5A1 QTL explained up to 20.8% and 5A2 up to 25.7% of FHB phenotypic variance, suggesting a moderate effect of these QTL. The same QTL were also reported in a backcross recombinant inbred line population from *T. turgidum* ssp. *dicoccum* line BGRC3487 and a derivative of DT696 (DT735)^5^. The QTL interaction analysis of the DT707 × DT696 population indicated the presence of an additive effect between the 5A1 and 5A2 QTL^2^. The 5A1 QTL co-located with a moderate plant height QTL and 5A2 with a major maturity QTL, providing supporting evidence for associations between these developmental traits and FHB resistance^2^. Three hypotheses are postulated for the co-localization of 5A1 with plant height and 5A2 with maturity QTL. FHB resistance may be the result of disease escape in taller and later-maturing plants as a consequence of height and maturity genes per se. A second possibility is that height or maturity loci have both disease escape and physiological FHB resistance effects, in other words there is a pleiotropic effect of height and maturity genes on FHB resistance. Alternatively, linkage between FHB resistance genes and genes for the developmental traits exist within the QTL intervals. Effective utilization of these QTL in breeding programs and the identification of predictive markers for marker-assisted selection (MAS) will depend on deciphering the type of association with plant height and maturity.

The association of FHB resistance QTL with plant height and maturity is not new. Previous studies highlighted a negative correlation between plant height and FHB severity^6,7^. The association of an FHB resistance QTL with the major effect plant height gene *Rht-B1*^8^ suggested the contribution of the dwarfing allele of *Rht-B1* to FHB susceptibility^9,10^. Buestmayr *et al*.^11^ found an FHB resistance QTL on chromosome 5A from *T. macha* co-located with the Q-gene that controls plant height, and spike traits including anthesis date, and spike density and length. The FHB resistance has also been considered pleiotropic to anther extrusion as the effect on resistance because of the co-localization of *Qfhs.ifa-5AS*, a FHB resistance QTL from hexaploid wheat line Sumai 3 on chromosome 5A, with an anther extrusion QTL^9,12,13^. These studies further emphasize the importance of greater understanding the relationship of FHB resistance and developmental traits through high-resolution mapping of FHB resistance QTL, designing markers for MAS, and the effective utilization of these QTL for developing resistant cultivars. A recent fine mapping effort that projected the interval of *Qfhs.ifa-5AS* on the International Wheat Genome Sequencing Consortia (IWGSC) Refseq v1.0^13^ along with the high-resolution mapping of the DT707 × DT696 population^2^ proposed the co-localization of *Qfhs.ifa-5AS* with 5A1 QTL.

The validation of QTL in breeding lines with relatively uniform plant height and maturity may enable teasing apart the contribution of disease escape mediated by tall alleles of plant height QTL from the potential physiological resistance conferred by the 5A1 and late alleles of maturity from the 5A2 QTL. Since its introduction as a source of moderate FHB resistance in the durum wheat breeding program at the Swift Current Research and Development Centre of the Agriculture and Agri-Food Canada (AAFC SCRDC), DT696 and its derivatives have been used as parents in several hundred crosses. This has provided an opportunity for the application of a genome wide association study (GWAS) for improving mapping resolution of FHB resistance QTL through the identification of novel recombination events at the 5A1 and 5A2 loci. It has also provided the opportunity for validating the 5A1 and 5A2 QTL and deciphering the type of the association of FHB resistance with plant height and maturity reported repeatedly in previous studies^2,8,11,14^. GWAS has been successfully used for the identification and mapping of FHB resistance QTL in a durum wheat diversity^15^ and a durum wheat breeding panel^16^. Thus, GWAS is valuable for validating and high-resolution mapping of the FHB resistance QTL previously identified through bi-parental mapping such as the 5A1 and 5A2 QTL.

High-resolution mapping of FHB resistance QTL is difficult because of the small effect of resistance loci with quantitative expression, variable expression across environments, their epistasis interaction with other loci, and their interactions with genetic background and developmental traits. To date, only resistance genes associated with *Fhb1*, an FHB resistance QTL on chromosome 3BS present in the hexaploid wheat line Sumai 3 have been identified^17–19^. Candidate resistance genes have been proposed for Sumai 3 FHB resistance loci *Qfhs.ifa-5A*, on chromosome 5A^20^ and *Fhb*2 on chromosome 6B^21^, and a Wuhan-1 resistance QTL on chromosome 2D^22^. High-resolution mapping of FHB resistance QTL in DT696 will contribute to the design of predictive markers for MAS, which will allow their rapid and effective utilization in breeding programs.

The objectives of this study were to evaluate lines derived from DT696 from the AAFC SCRDC breeding program for FHB resistance, to sample recombination in the chromosome 5A region for high-resolution mapping of the FHB resistance QTL, to further elucidate the genetic relationship of height and maturity to FHB resistance, and to identify additional loci contributing to FHB resistance in the breeding lines. We genotyped 401 DT696 derivatives available from the AAFC SCRDC durum wheat breeding program with the 90 K iSelect SNP assay^23^. A subset of 223 lines representing the recombinants at the 5A1 and 5A2 QTL were phenotyped in multiple environments and subjected to GWAS using the 90 K SNPs. This reduced the size of 5A2 QTL interval to 15.8 Mbp. An FHB resistance QTL in a low recombination region of chromosome 6B was also identified. A panel of 523 contemporary breeding lines from the AAFC SCRDC breeding program with relatively uniform plant height and maturity was genotyped with SNP markers associated with the 5A2 interval, assessed for FHB resistance and subjected to haplotype-FHB trait association analysis. Significant association between markers with the DT696 allele and FHB traits validated the markers for MAS and demonstrated that the contribution of 5A2 QTL to FHB resistance is partially independent of plant height and maturity. DT696 haplotype and novel recombination events were present in 1022 lines of the Global Tetraploid Wheat Collection (GTWC). The novel recombination events constitute valuable resources for further refining the genetic interval and identifying candidate gene. The presence of the DT696 haplotype in the diversity panel will provide opportunity for the utilization of the resistance in other regions of the world from which the diversity panel originated.

## Results

### Trait variation and correlation

Variance analysis was conducted for the FHB traits (incidence, severity and index) as well as plant height and maturity for 223 DT696 derivatives phenotyped in Morden and Brandon FHB nurseries in Manitoba, Canada in 2016 and 2017. The line variation for FHB severity and index, and plant height was significant in all environments except at Morden in 2016 (Supplementary Table S1 online), whereas the line variation for FHB incidence was non-significant across all environments. The line variance for maturity was non-significant across environments. Considering the co-localization of plant height and maturity with FHB resistance QTL reported in the previous study^2^, covariance analysis conducted with either plant height or maturity assigned as a covariate to test the dependency of FHB resistance expression on these traits. The genotypic variation for FHB severity and index remained significant when either plant height or maturity was assigned as covariate.

All FHB trait-environment combinations were significantly correlated with plant height (Supplementary Table S2 online), except at Morden in 2016 where only FHB incidence was correlated. There were low to moderate negative correlations between FHB traits and plant height, reflecting slightly lower FHB symptoms in taller plants. The correlations were slightly higher at Brandon than Morden. Correlation with plant maturity was significant for all the FHB traits measured across environments. There were low positive correlations between FHB traits and maturity, reflecting slightly lower FHB symptoms in later maturing plants.

**Figure 1:**
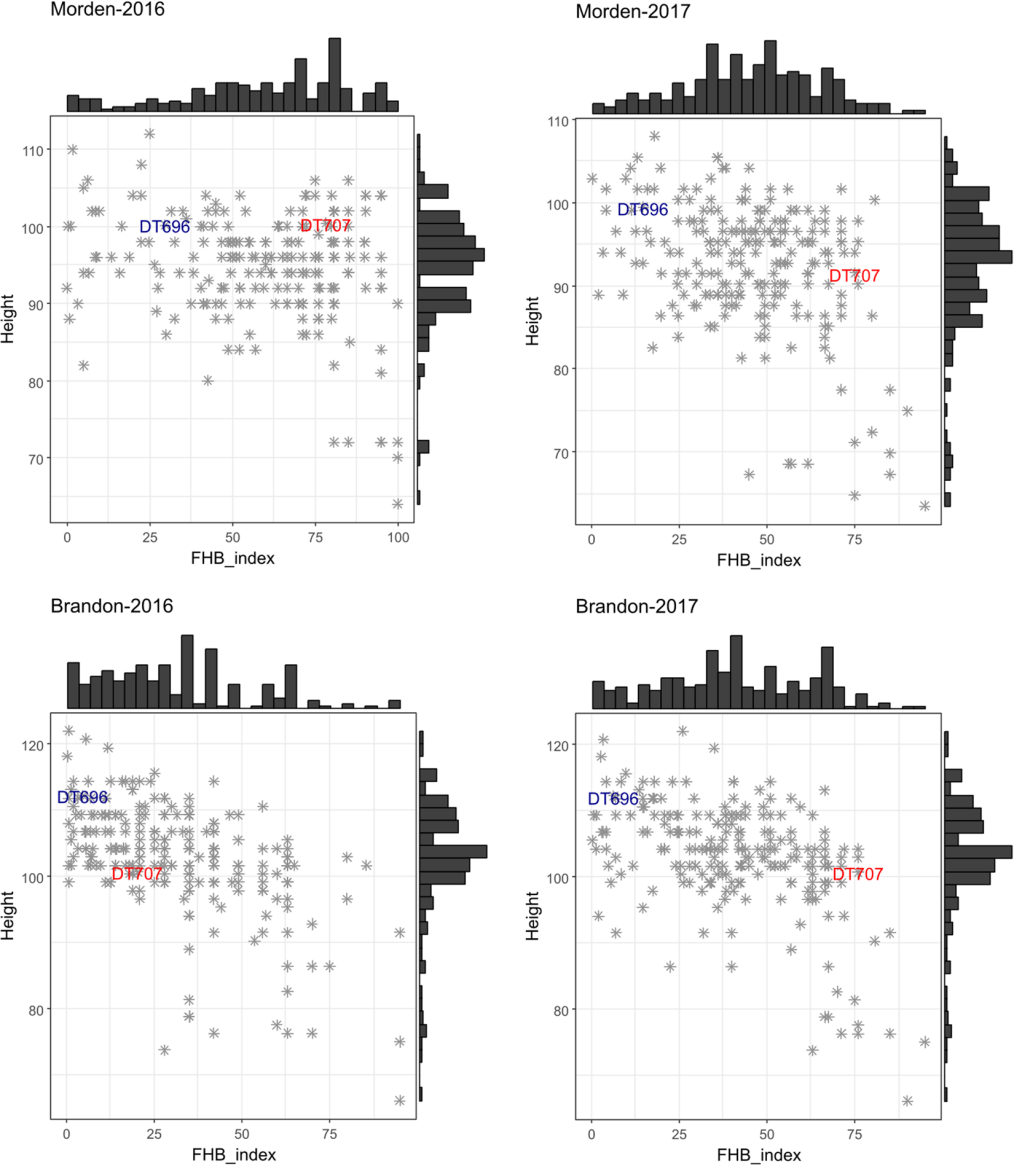
Phenotypic distribution of FHB index and its association with plant height for 223 DT696 derivatives phenotyped in field nurseries located at Morden and Brandon in 2016 and 2017. Each asterisk in the dot plots represents a single DT696 derivative line. The position of checks is marked in the histogram in blue font for DT696 and red font for DT707. Bars of the histograms on the top and right-side of the plots show the number of lines in each phenotypic category.

**Figure 2:**
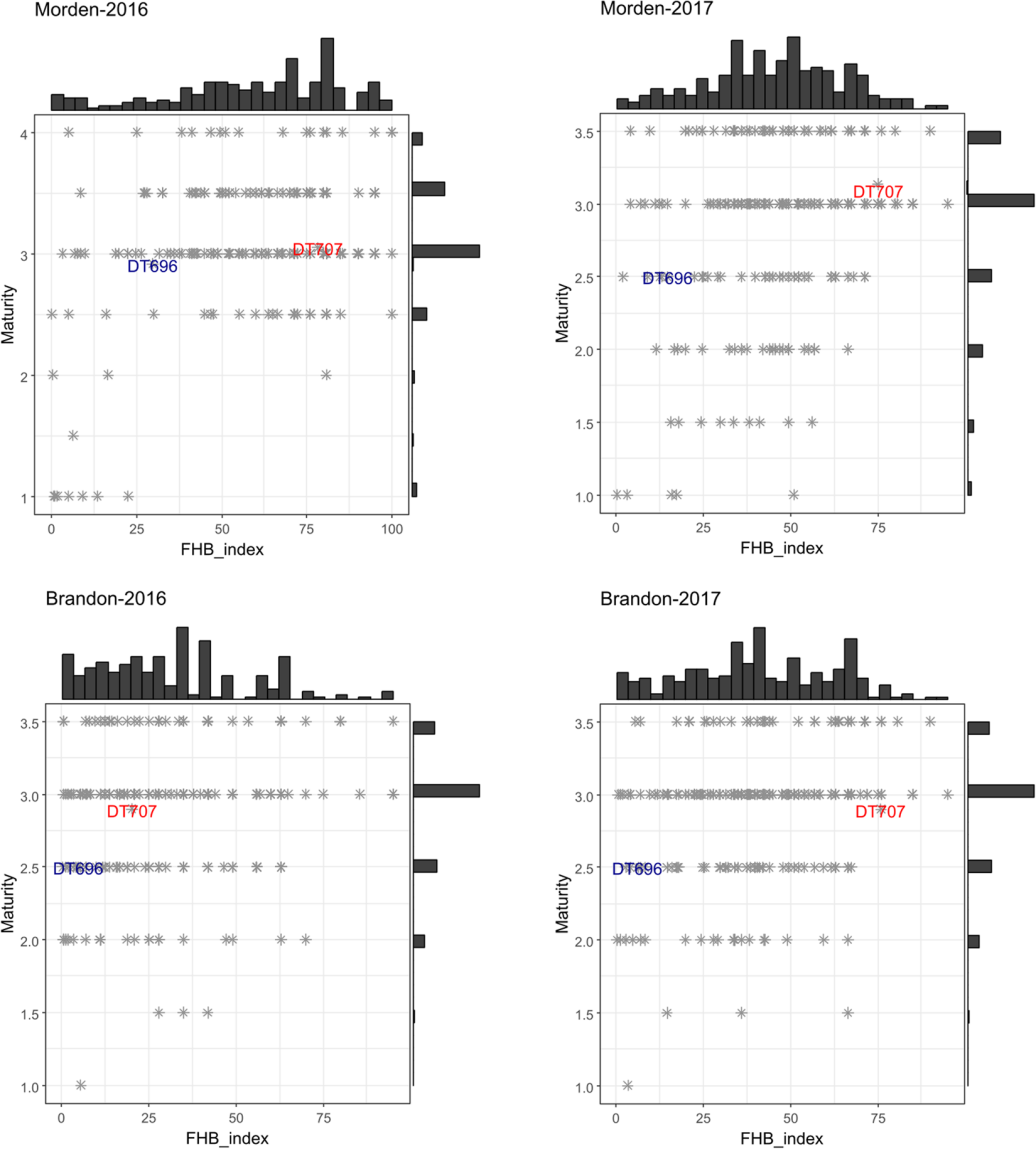
Phenotypic distribution of FHB index and its association with plant maturity for 223 DT696 derivatives phenotyped in field nurseries located at Morden and Brandon in 2016 and 2017. Each asterisk in the dot plots represents a single DT696 derivative line. The position of checks is marked in the histogram in blue font for DT696 and red font for DT707. Bars of the histograms on the top and right-side of the plots show the number of lines in each phenotypic category. Maturity was measured on a scale of 1–4 with 1 representing latest maturity and 4 representing earliest maturity.

A high level of disease occurred at Morden in 2016 with most of lines having 50% or higher FHB index (Fig. 1), whereas most of the lines had an FHB index lower than 50% at Brandon in 2016. The FHB index of trials conducted in 2017 at Morden and Brandon were similar and most lines had an FHB index of 25–75%. DT696 had a lower FHB index than the susceptible check DT707 in all environments. DT696 was often 10 cm taller than DT707, except at Morden in 2016 where they had nearly identical plant height. Most DT696 derivatives had a plant height of 85–105 cm at Morden while 95–115 cm at Brandon, reflecting the effects of environment on expression of this trait. The tallest and latest-maturing lines in all environments had often the lowest FHB index; however, moderate and rarely low FHB index (<10%) was observed in semi-dwarf (90–100 cm tall) and early-maturing (maturity score 3 or higher) lines (Fig. 1 & 2).

### Linkage disequilibrium decay and GWAS

The trend of linkage disequilibrium (LD) decay calculated from pair-wise SNP LD squared allele frequency correlation (*r*^2^) plotted against the pair-wise genetic distance on average was 1.6 cM in the DT696 derivative lines (Supplementary Fig. S1 online). Analysis of population structure indicated the presence of at least four sub-populations with several admixtures in each subpopulation (Supplementary Fig. S2 online).

**Figure 3:**
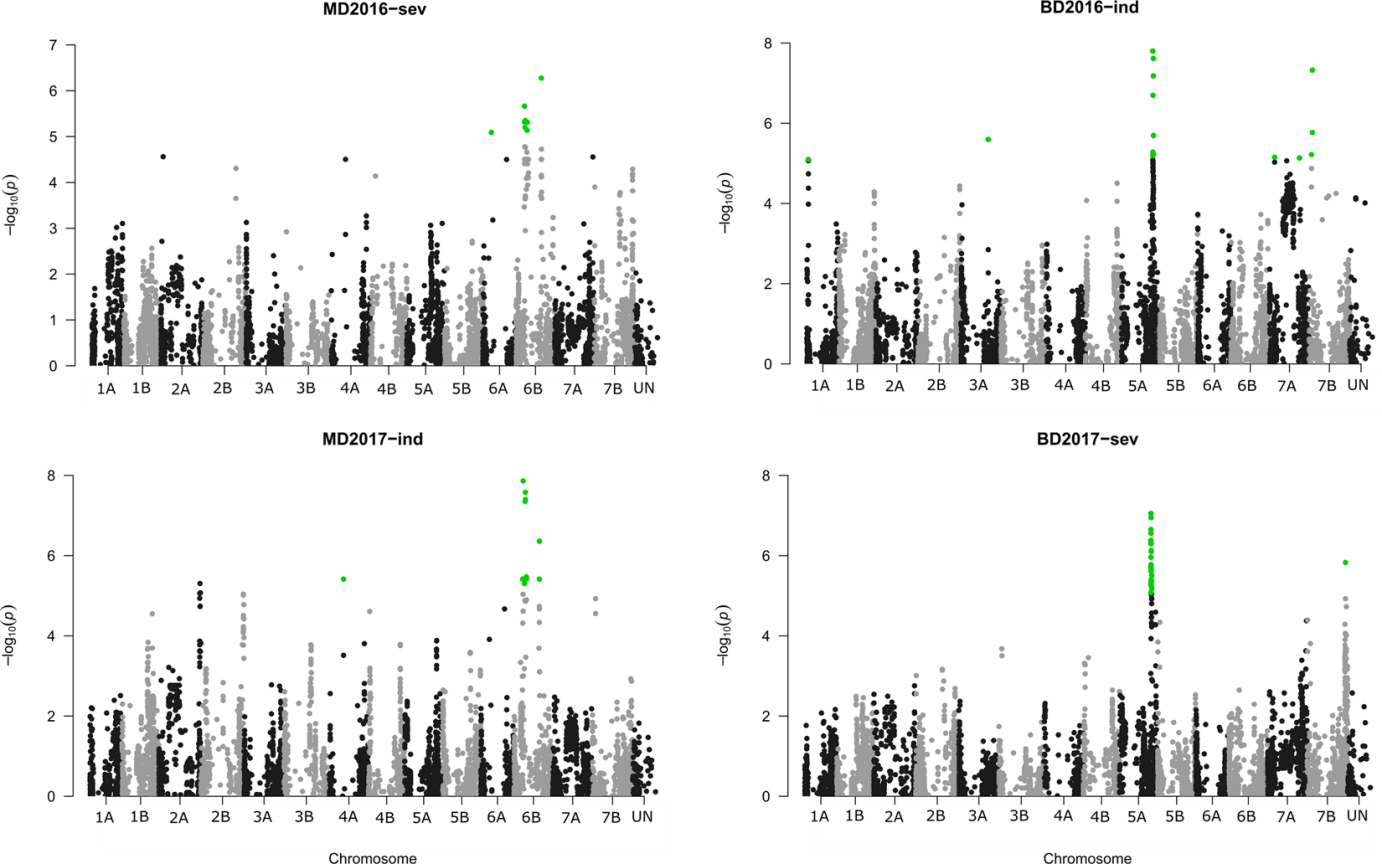
Genome-wide association of Single Nucleotide Polymorphisms (SNPs) with Fusarium head blight traits (severity [sev] and index [ind]) of the DT696 derivative field trials at Morden (MD) and Brandon (BD) locations in 2016 and 2017. Significant SNPs are shown as green dots and are determined based on Bonferroni adjusted *P* value which was equal to a marker-wise *P* value of 8.25 × 10^−6.^ The y-axis represents the *P* value of the SNP–trait association on a − log_10_ scale and the x-axis represents the physical position of markers on 14 durum wheat chromosomes obtained from anchoring SNPs to the International Wheat Genome Sequencing Consortium (IWGSC) RefSeq v.1.0 genome assembly^37^. SNPs belonging to category UN are within scaffolds not assigned to the chromosomes in the IWGSC Refseq v1.0 assembly. Only environments and FHB traits with significant *P* values are presented as Manhattan plots, out of the GWAS results presented in Supplementary file 1.

GWAS analysis identified significant association between one SNP on chromosome 6A and eight SNPs on chromosome 6B with FHB severity in 2016 as well as one SNP on chromosome 4A and 13 SNPs on chromosome 6B with FHB index in 2017 at the Morden nursery (Supplementary file 1 online and Fig. 3). Three of the SNPs on chromosome 6B were associated with FHB traits at the Morden nursery in both 2016 and 2017 (Supplementary file 1 online), suggesting a stable expression over years of the FHB resistance locus spanned by these three SNPs. Significant association was also identified between one SNP on chromosome 1A, nine SNPs on 5A, two SNPs on 7A and three SNPs on 7B for FHB index in 2016, and between 28 SNPs on chromosome 5A and one SNP on 7B for FHB severity in 2017 at the Brandon nursery. Four of the SNPs on chromosome 5A were associated with FHB traits at Brandon in both 2016 and 2017, suggesting a stable expression over years of the FHB resistance locus spanned by these four SNPs. None of these loci remained significant across locations, suggesting a genotype × location interaction.

**Figure 4:**
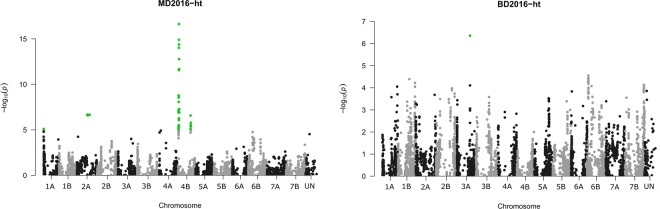
Genome-wide association of Single Nucleotide Polymorphisms (SNPs) with plant height (ht) of the DT696 derivative field trials at Morden (MD) and Brandon (BD) locations. Significant SNPs are shown as green dots and are determined based on Bonferroni adjusted *P* value which was equal to a marker-wise *P* value of 8.25 × 10^−6^. The y-axis represents the *P* value of the SNP–trait association on a − log_10_ scale and the x-axis represents the physical position of markers on 14 durum wheat chromosomes obtained from anchoring SNPs to the International Wheat Genome Sequencing Consortium (IWGSC) RefSeq v1.0 genome assembly^37^. SNPs belonging to category UN are within scaffolds not assigned to the chromosomes in the IWGSC Refseq v.1.0 assembly. Only environments and FHB traits with significant *P* values are presented as Manhattan plots, out of the whole GWAS results presented in Supplementary file 1.

Single nucleotide polymorphisms associated with plant height were revealed on chromosomes 1A (two SNPs), 2A (four SNPs) and two physically distant loci on chromosome 4B (23 and six SNPs) in the Morden 2016 environment, and on chromosome 3A (one SNP) at the Brandon location of the same year (Supplementary file 1 online, Fig. 4). The two SNPs on chromosome 1A formed a 0.1 Mbp interval that was physically distant from the SNP associated with FHB index at Brandon in 2016. Of the SNPs associated with plant height at Morden in 2016, *Tdurum_contig*2*7834_*260 was located within the major wheat dwarfing gene *Rht-B1* (TraesCS4B01G043100). A two tailed *t*-test of plant height between lines carrying alternate alleles at *Tdurum_contig*2*7834_*260 was significant (*P* = 2.78E-5) and lines carrying the dwarfing allele (17 out of 223 lines) were on average 10.6 cm shorter than those with the alternate allele (Supplementary Fig. S3 online). The difference between the FHB index of lines carrying the alternate alleles was also significant (*P* = 0.02).

**Figure 5:**
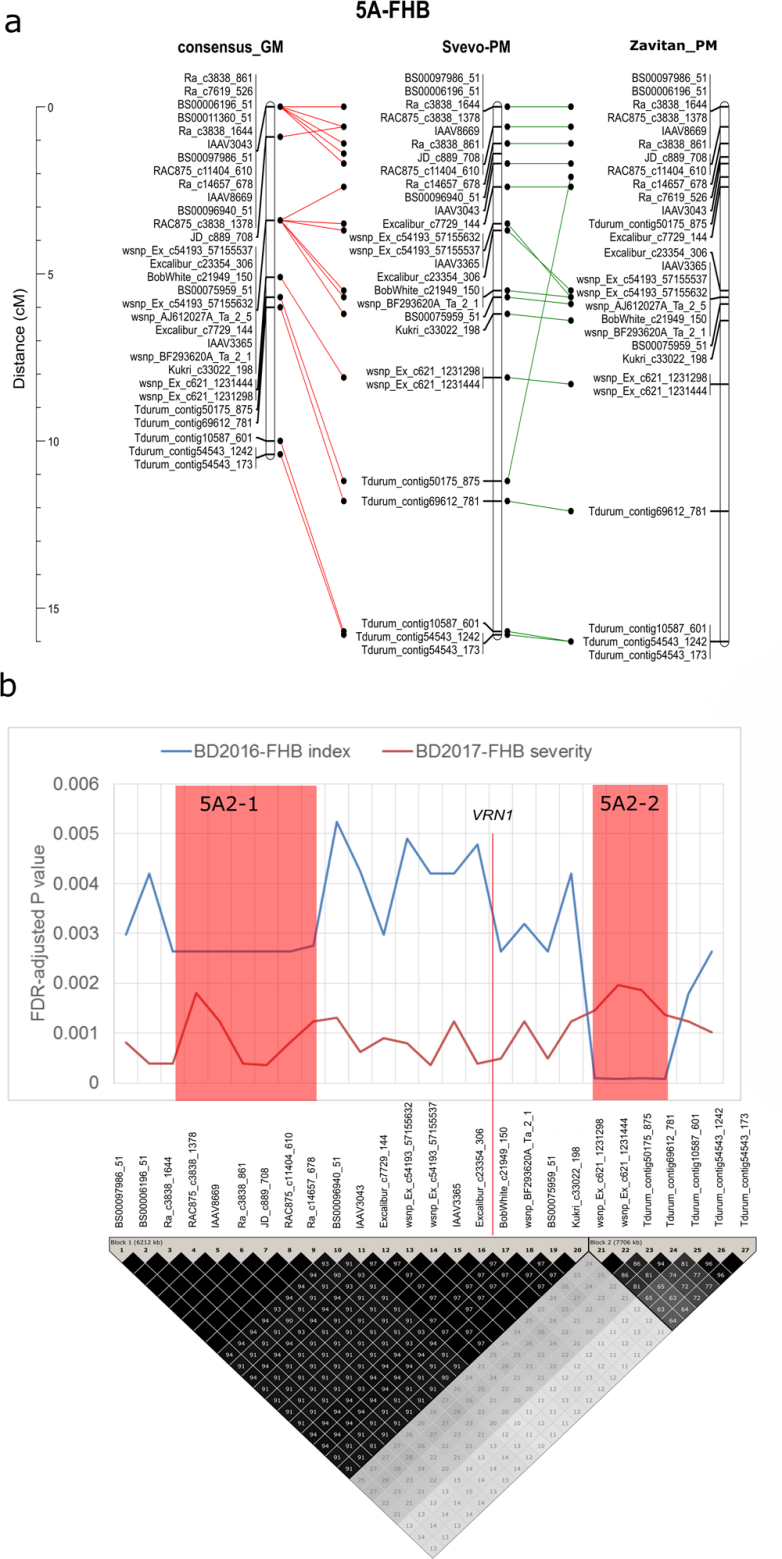
The genetic versus physical locations (**a**), and false discovery rate (FDR) adjusted *P* value and pair-wise linkage disequilibrium (LD) squared allele frequency correlation (*r*^*2*^) (**b**) of Single Nucleotide Polymorphisms (SNPs) on chromosome 5A that were associated with Fusarium head blight traits (severity and index) of DT696 derivatives at the Brandon (BD) environment. Chromosomal map position of markers on the consensus genetic map (GM) of Maccaferri *et al*.^24^ and the corresponding physical distance on durum wheat cv. Svevo^38^ and wild emmer wheat accession Zavitan^39^ reference physical maps (PM) are compared (a). The genome-wide association study (GWAS) FDR adjusted *P* value of each SNP significantly associated with FHB traits are projected over its LD *r*^*2*^ values (b). The vertical red line shows the position of the major vernalisation gene *VRN1* (TraesCS5A01G391700) and the areas shaded in red are the locations of the 5A2–1 and 5A2–2 intervals that had lower FDR adjusted *P* value than the surrounding markers for FHB index at Brandon in 2016. The order of SNPs in panel (b) is inferred from their position on cv. Svevo physical map.

The SNPs on chromosome 5A that were significantly associated with FHB index and severity spanned a 10 cM interval (Fig. 5a) on the consensus genetic map of Maccaferri *et al*.^24^. Anchoring these markers to the durum wheat cv. Svevo and wild emmer wheat accession Zavitan physical maps partially supported the order of markers on the consensus genetic map and confirmed the association of the SNPs with a single physical interval. The GWAS false discovery rate (FDR) adjusted *P-*value varied between the SNPs within this interval and was often lower in the 2017 than the 2016 environment (Fig. 5b). The FDR adjusted *P*-value of SNPs within a 1.1 Mbp interval between SNPs *Ra_c3838_1644* and *Ra_c14657_678* (5A2–1) and a 3.7 Mbp interval between SNPs *wsnp_Ex-c6*2*1_1*231298 and *Tdurum_contig_6961*2*_781* (5A2–2) was lower (more significant) than their flanking markers in the 2016 environment. A large drop in LD *r*^2^ at *wsnp_Ex-c6*2*1_1*231298 suggested the presence of a frequent crossover in the DT696 derivatives at this locus. The physical position of the vernalisation gene *VRN1* (TraesCS5A01G391700) inferred from the IWGSC Refseq. v1.0 annotation was between the 5A2–1 and 5A2–2 intervals (Fig. 5b).

**Figure 6:**
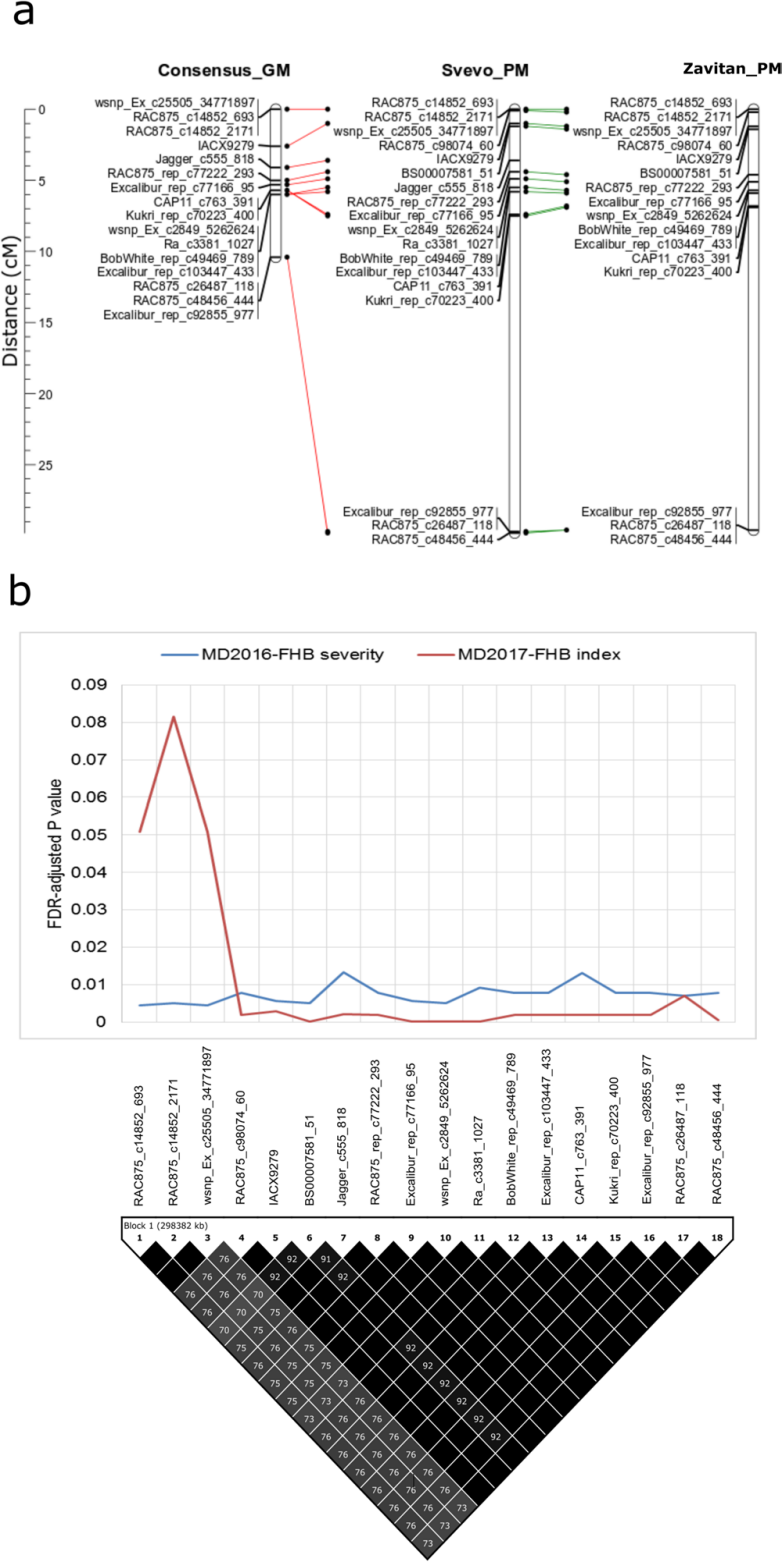
The genetic versus physical locations (**a**), and false discovery rate (FDR) adjusted *P* value and pair-wise linkage disequilibrium (LD) squared allele frequency correlation (*r*^*2*^) (**b**) of Single Nucleotide Polymorphisms (SNPs) on chromosome 6B that were associated with Fusarium head blight traits (severity and index) of DT696 derivatives at the Morden (MD) environment. Chromosomal map position of markers on tetraploid consensus genetic map (GM) of Maccaferri *et al*.^24^ and their physical distance on durum wheat cv. Svevo^38^ and wild emmer wheat accession Zavitan^39^ reference physical maps (PM) are compared in panel (a). In panel (b), the GWAS FDR adjusted *P* value of each marker significantly associated with FHB traits is projected over the LD *r*^*2*^ values. The order of SNPs in panel (b) is inferred from their position on the cv. Svevo physical map.

Single Nucleotide Polymorphisms significantly associated with FHB traits on chromosome 6B spanned a 10.4 cM interval on the tetraploid wheat consensus genetic map of Maccaferri *et al*.^24^. Anchoring of the SNPs to the durum wheat cv. Svevo and wild emmer wheat accession Zavitan physical maps confirmed the order of markers on the consensus genetic map and confirmed that the markers belong to a single physical interval (Fig. 6a). SNPs had similar FDR adjusted *P*-values over environments, except for the interval between SNPs *RAC875_c1485*2*_693* and *wsnp_Ex_c*2*5505_34771897* on the left boundary of the interval that had non-significant FDR adjusted *P*-values (>0.05) at Morden in 2017 (Fig. 6b). A lower LD *r*^2^ value of *wsnp_Ex_c*2*5505_34771897* than the proximal markers reflected the presence of a recombination event at this locus in the DT696 derivatives.

**Figure 7:**
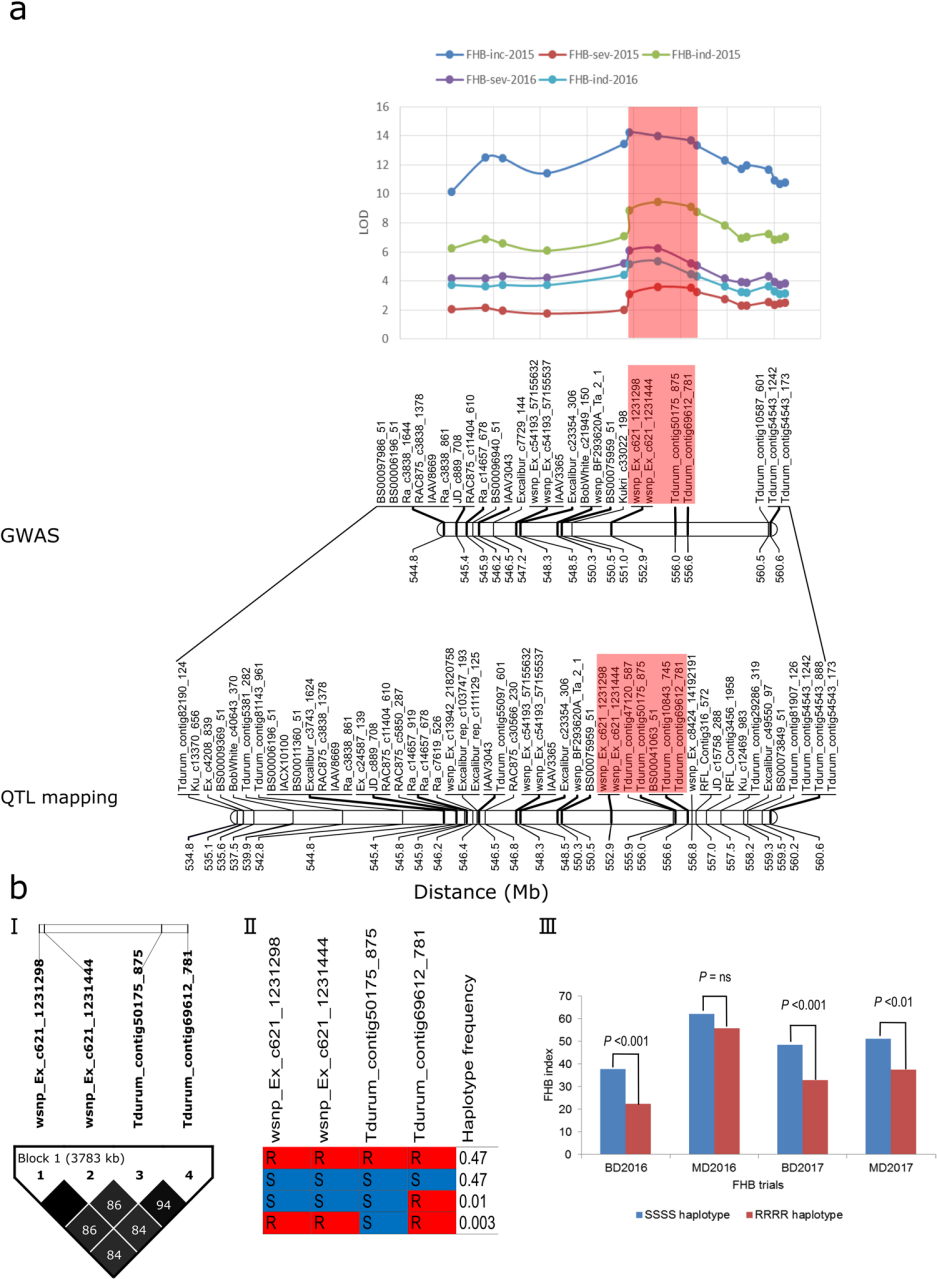
Fusarium head blight resistance QTL interval on chromosome 5A (5A2) inferred from high-density QTL mapping of the DT707 × DT696 population^2^ versus the genome-wide association study (GWAS) of DT696 derivatives (**a**) and haplotypes captured in DT696 derivatives in an interval between single nucleotide polymorphisms (SNPs) *wsnp_Ex-c621_1231298* and *Tdurum_contig_69612_781* that had the most significant false discovery rate (FDR) adjusted *P* value in GWAS (**b**). The top chart in panel (**a**) shows the logarithm of odds (LOD) values of the QTL mapping of the DT707 × DT696 population^2^. The physical location (on cv. Svevo physical map^38^) of the QTL interval inferred from QTL mapping of the DT707 × DT696 population is compared with GWAS of the DT696 derivatives. Areas shaded in red show the interval with the lowest FDR adjusted *P* value in the GWAS analysis (5A2–2 interval). Panel (b) shows the pair-wise linkage disequilibrium (LD) squared allele frequency correlation (*r*^*2*^) value of SNPs (I), haplotypes present in the derivatives in the 5A2–2 interval (II) and results of two tailed *t*-tests between FHB index rated at Morden and Brandon locations in 2016 and 2017 of lines carrying DT696 (R) versus DT707 (S) alleles at this interval (III).

Comparing the physical positions of SNPs spanning the interval on chromosome 5A inferred from QTL mapping of the DT707 × DT696 population^2^ and GWAS of the derivatives suggested their association with the same physical interval (Fig. 7a). The interval inferred from QTL mapping of the DT707 × DT696 population was 25.8 Mbp, 15.8 Mbp of which overlapped with significant markers identified by GWAS. SNPs within the 5A2–2 interval had slightly higher logarithm of odds (LOD) values than the other SNPs in the QTL mapping analysis (Fig. 7a top panel). No recombination (LD *r*^2^ = 1) was detected in DT696 derivatives between SNPs *wsnp_Ex-c6*2*1_1*2*31*2*98* and *wsnp_Ex-c6*2*1_1*2*31444*, however, rare recombination events were detected between the other SNPs spanning the 5A2–2 interval (Fig. 7b-I). Recombinants formed 1% of the derivative population at the 5A2–2 interval, and 94% of lines had either the DT696 (resistant) or the DT707 (susceptible) haplotype (Fig. 7b-II). Lines with the resistant and susceptible haplotypes in the 5A2–2 interval were significantly different (two-tailed *t*-test *P* < 0.05) in FHB index at Brandon in 2016 and 2017, and at Morden in 2017 but not in 2016 (Fig. 7b-III). The difference between the FHB index of lines carrying the resistant and susceptible haplotypes at the 5A2–2 interval was larger at the Brandon environment and reached to 20% on average at this location in 2016.

### Validation of SNPs for marker-assisted selection

**Figure 8:**
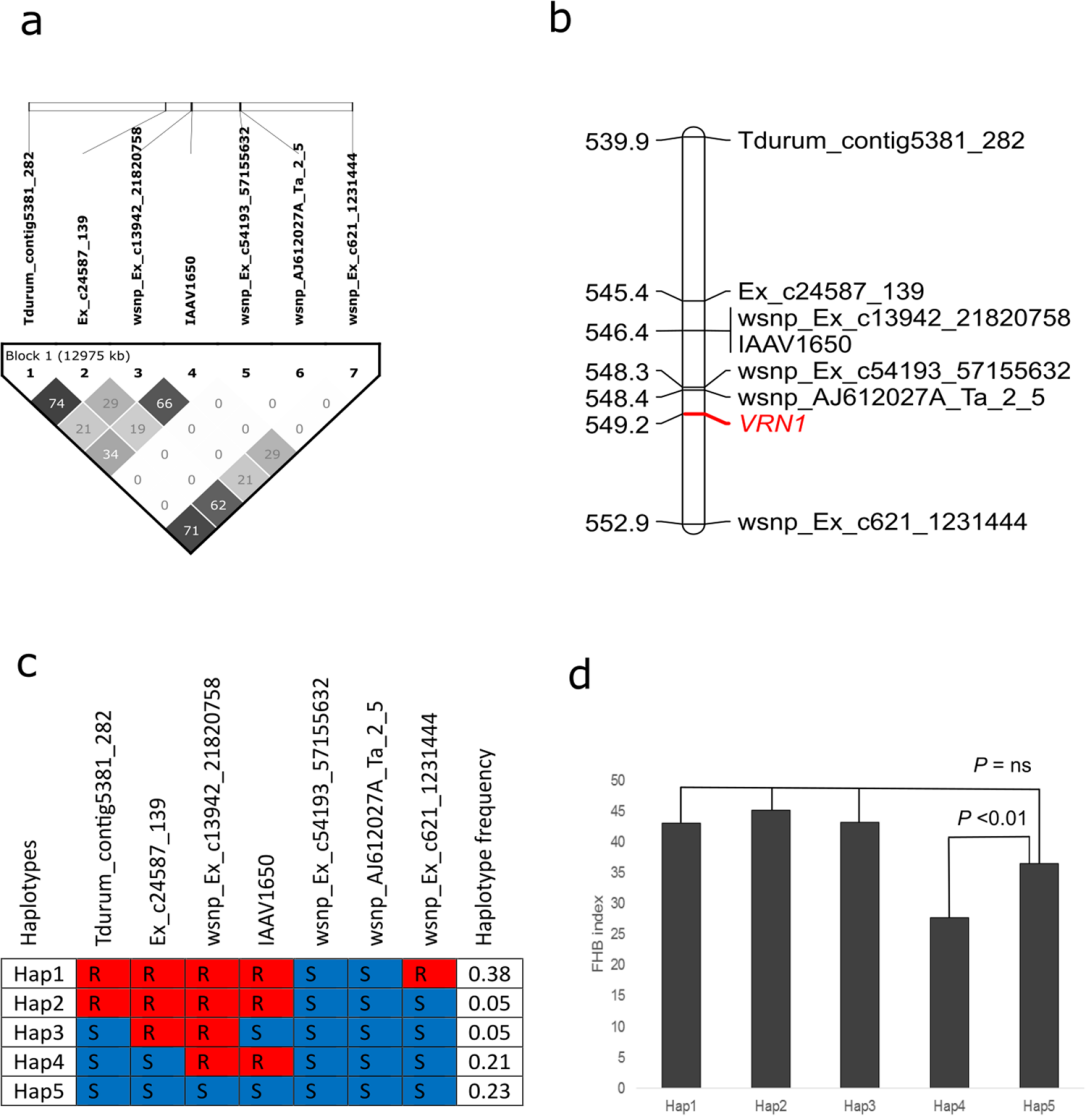
Validation of single nucleotide polymorphisms (SNPs) associated with the Fusarium head blight QTL on chromosome 5A (5A2) in 523 contemporary breeding lines. SNPs selected for genotyping and their pair-wise linkage disequilibrium (LD) squared allele frequency correlation (*r*^*2*^) are shown in panel (**a**). The physical location of SNPs on the durum wheat cv. Svevo assembly^38^ and their physical distance from the vernalisation gene *VRN1* (TraesCS5A01G391700) is shown in panel (**b**). Haplotypes detected in the breeding population are presented in panel (**c**) and the results of the two-tailed *t*-test between FHB indexes of these haplotypes are presented in panel (**d**). R in panel (c) stands for resistance alleles of DT696 and S for susceptibility alleles of DT707.

A few SNPs spanning the 5A2 FHB resistance QTL interval inferred from the QTL mapping of the DT707 × DT696 population were used to genotype 523 contemporary breeding lines developed at AAFC SCRDC. According to the physical positions, the interval formed by these SNPs covered the entire 5A2–1 and a portion of 5A2–2 interval. All lines carried the DT707 allele at the interval between SNPs *wsnp_Ex_c54193_5715563*2 and *wsnp_AJ61*2*0*2*7A_Ta_*2*_5* that was 0.8 Mbp away from *VRN1* (Fig. 8a,b,c). The phenotyping was conducted at two FHB nurseries, where 75% of lines spray inoculated at the nursery in Carman, Manitoba and the remaining 25% were phenotypes at the Brandan nursery with corn spawn inoculation. When the FHB index of haplotypes carrying cross-overs (Hap 1–4) was compared with that of the haplotype carrying the DT707 (susceptibility) alleles in the interval (Hap5), only Hap4 that had DT696 (resistance) alleles in an interval between SNPs *wsnp_Ex_c13942_21820758* and *IAAV1650* and susceptibility allele at other loci had significantly lower FHB index (Fig. 8d). This haplotype was present in 21% of the contemporary breeding lines. The FHB index of lines with Hap 4 was on average 9% lower than those with Hap 5. To test if the inoculation method altered the expression of QTL, we conducted a two-tailed *t*-test between FHB indexes of the haplotypes after splitting the data based on the inoculation methods. Hap 4 remained significantly different from Hap 5 at both Carman (spray inoculation, *P* = 0.00012) and Brandon (corn-spawn inoculation, *P* = 0.00282) nurseries, while the difference among other haplotypes remained insignificant.

### Haplotype and recombination breakpoints in the Global Tetraploid Wheat Collection (GTWC)

**Figure 9:**
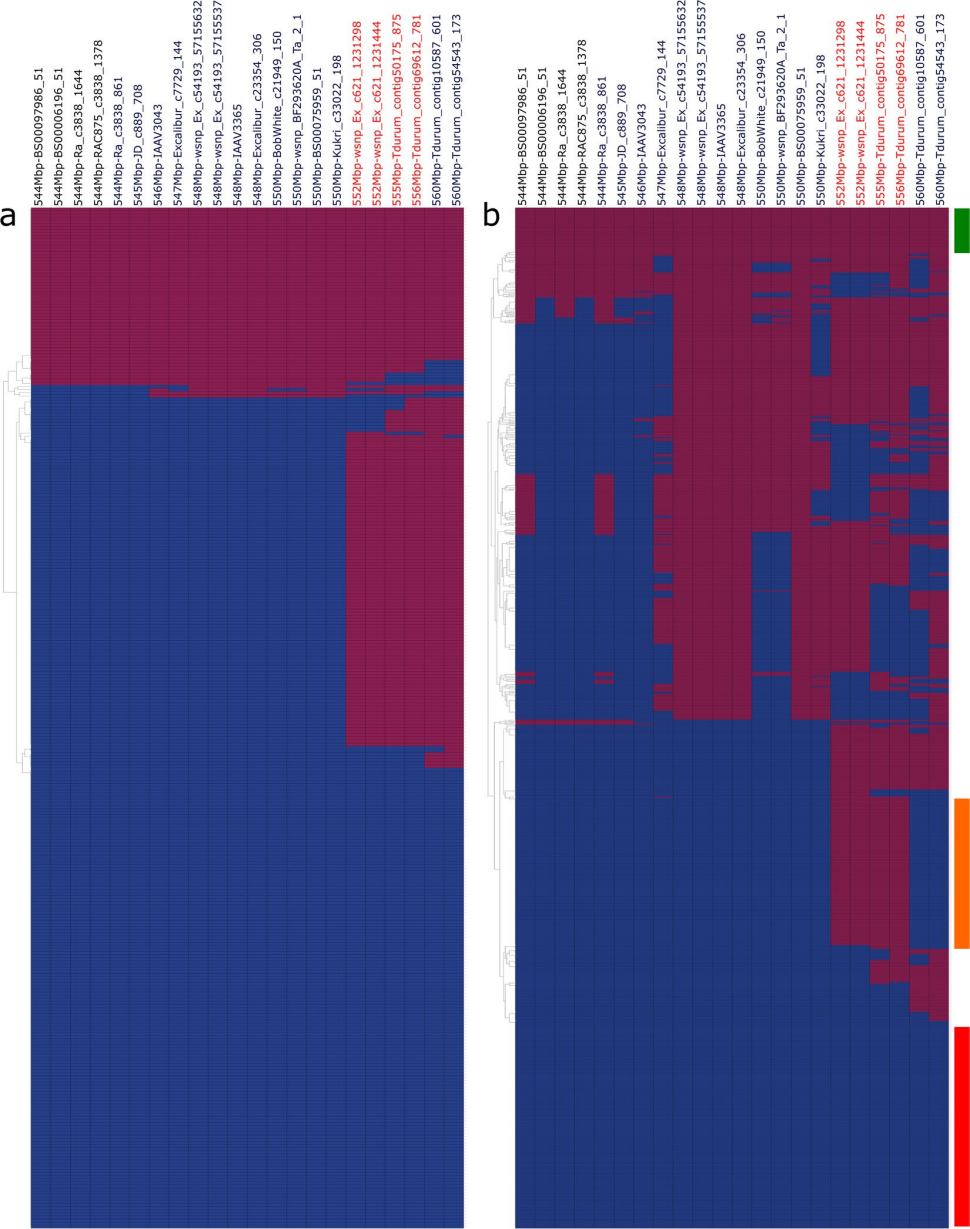
Haplotype of the DT696 derivatives (**a**) and Global Tetraploid Wheat Collection (**b**) at the 15.8 Mb interval associated with the 5A2 FHB resistance QTL. The DT696 allele is shown with red and the DT707 with blue tiles. Single Nucleotide Polymorphism (SNP) marker names in red are associated with the 5A2–2 interval. The bars on the right side of panel (b) show lines of Global Tetraploid Wheat Collection with haplotypes of DT707 (red bar), or DT696 (green bar) and a set of 149 lines (orange bar) with DT696 alleles only at the 5A2–2 interval at physical position of 552–556 Mbp between SNPs *wsnp_Ex_c621_1231298* and *Tdurum_contig69612_781*. The bar color represents the color chosen to group lines in Supplementary file 2 online.

Within DT696 derivatives, almost half of the lines carried the DT707 haplotype and the rest carried either the resistant or a recombinant haplotype (Fig. 9a) at 5A2 QTL interval. The GTWC landraces with the DT696 haplotype at the 5A2 QTL interval belonged to geographically distinct origins including North America, North Africa, West, Centre and South Asia, and South Europe (Supplementary file 2 online). The most common recombination breakpoint in the DT696 derivatives was at physical position 550–552 Mbp on the cv. Svevo physical map (Fig. 9a), between SNPs *Kukri_c33022_198* and *wsnp_Ex_c621_1231298*; this was also a common breakpoint in the lines of GTWC (Fig. 9b). The GTWC also carried a number of unique haplotypes that were not present in the DT696 derivatives, more proximal at 546–548 Mbp.

We detected 149 lines in the GTWC with only DT696 alleles in the 5A2–2 interval at physical position of 552–556 Mbp between SNPs *wsnp_Ex_c621_1231298* and *Tdurum_contig69612_781* (Fig. 9b; Supplementary file 2 online). When the origin of GTWC lines with only DT696 alleles in the 5A2–2 interval was examined, 93% were landraces from Eastern and Northern-Africa.

## Discussion

GWAS analysis of DT696 derived lines indicated a location-dependent expression of FHB resistance loci, with SNPs within the 5A-2 QTL only associated with resistance at Brandon and those on chromosome 6B only at Morden. The expression of these QTL was consistent over years, suggesting that location was a larger source of environmental variation than year. This could be partially related to difference in disease pressure between Morden and Brandon which, for example, was very high in 2016 where the FHB index of the susceptible check (DT707) was approximately 50% higher at Morden than Brandon. The effect of disease pressure on the expression of FHB QTL was observed by Clark *et al*.^25^ who indicated that the effectiveness of *Fhb1* in reducing FHB symptoms and deoxynivalenol (DON) was lowered by high disease pressure, which could be similar to what we observed for 5A-2. Moreover, a genotype by environment interaction was expected based on the moderate heritability of FHB resistance in DT696 which was previously inferred from phenotyping the DT707 × DT696 population at multiple field nurseries including Morden where the DT696 derivatives were phenotyped in the present study^2^. Such a genotype × location interaction had lower impact on the expression of 5A2 QTL in the DT707 × DT696 population than the derivatives as the 5A2 QTL was consistently expressed over environments in the previous mapping study on the DT707 × DT696 population^2^, but was expressed only at Brandon location for the derivatives (Supplementary file 1 online). The 5A2 QTL was detected at the Morden FHB nursery in 2016 and 2017 for the DT707 × DT696 population with the percentage of phenotypic variation of 4–4.9% in 2016 and 6.3–10.9% in 2017 for various FHB traits. The association of 5A2 QTL with FHB traits might have fallen below the detection threshold of GWAS at Morden nursery due to the higher disease pressure, which could make the small differences between carrier and non-carrier lines undetectable.

The presence of DT696 derivative lines with equal and occasionally higher FHB resistance, but shorter and earlier maturity than DT696 suggests that the resistance may not be solely due to disease escape of the taller and later-maturing plants. Moreover, when plant height and maturity were assigned as covariates in the variance analysis of FHB trait means, the variance of lines remained significant, further supporting the minor effect of plant height and maturity on expression of FHB resistance in the derivatives. Nevertheless, moderate to low correlations of FHB traits with plant height and maturity indicated that FHB resistance is partially associated with the developmental traits in the DT696 derivatives. However, there is no evidence to suggest that the FHB resistance genes segregating in the population are inextricably associated with plant height and maturity. In fact, higher numbers of recombinants in the derivatives could allow the decay of linkage between genes for the developmental traits and those for FHB resistance within the 5A2 region, that was according to Sari *et al*.^2^, associated with both FHB resistance and plant maturity^2^. Such linkage decay helps to explain the rare occurrence of lines moderately resistant yet earlier maturing than DT696 within the DT696 derivative set. The association of developmental and FHB resistance genes at a single locus is not new. The co-localization of the plant height gene *Rht8* and an FHB resistance QTL on chromosome 2D was previously reported^26^. Subsequently, Handa *et al*.^14^ identified genes associated with DON degradation as the basis for FHB resistance in this interval, demonstrating that *Rht8* was not the sole contributor to FHB resistance. The availability of additional recombination events within the 5A2 QTL interval in the GTWC panel is an opportunity to validate further the possibility of a similar disassociation in the 5A2 QTL.

The SNP within *Rht-B1* (*Tdurum_contig27834_260*) was associated with plant height only at Morden in 2016. The effect of the locus partially explains the shorter plant height of the DT696 derivatives at this location compared to Brandon. Despite a relatively large association of *Tdurum_contig27834_260* (GWAS *R*^*2*^ = 0.29) with plant height, it was not detected as an SNP significantly associated with FHB susceptibility in the GWAS. This is different from the results of He *et al*.^10^ that suggested that the *Rht-B1* tall allele is associated with Type I FHB resistance (resistance to initial infection or incidence), reflecting lower FHB incidence in taller plants. Even though *Rht-B1* was not detected as an FHB resistance QTL in the GWAS, a two-tailed test found significant association between the SNP within this gene and FHB index at the Morden nursery in 2016 (Supplementary Fig. S3 online). The Bonferroni corrected *P* value that was used to identify SNPs associated with FHB in the GWAS analysis may be too stringent given the relatively low FHB variance (*P* = 0.02) between lines with alternative alleles of *Tdurum_contig27834_260*. It could also be a consequence of the low frequency (8%) of the dwarfing allele of *Rht-B1* in the derivative population. Nevertheless, the Type II resistance which is independent of *Rht-B1* seems to be the major determinant of FHB resistance in the derivatives. This is supported by the non-significant variance of lines for FHB incidence across all four environments and the identification of the 5A2 and 6B FHB resistance loci only for FHB severity and index ratings, but not for FHB incidence.

The presence of four sub-populations in the DT696 derivatives suggested the presence of substantial genetic variation in the population. This variation would have originated from genetic diversity of the founders used for crossing with DT696 (Supplementary file 3 online). The DT696 derivatives are lines that derive from 72 different crosses. Detection of the 5A2 FHB resistance QTL in this set of lines and in the DT707 × DT696 population suggests that it is consistently expressed in a relatively wide range of genetic backgrounds. Consistency of expression was also reported for *Fhb1*, which in a previous study was expressed in a wider range of winter wheat genetic backgrounds than the FHB resistance QTL *QFhs.nau-2DL*^25^. The consistency of expression in a diversity of genetic backgrounds improves the success of recovering the 5A2 FHB resistance QTL during breeding of elite durum germplasm and explains the success achieved with the improved FHB resistance in durum varieties, derived from DT696, such as Brigade, Transcend and CDC Credence^3,4^.

Improvement in mapping resolution over biparental QTL analysis was achieved with the DT696 derivatives as demonstrated by the interval being 10 Mbp shorter following the GWAS. This is likely due to a higher number of recombination events in the derivatives than the bi-parental population used for QTL mapping. The decay of linkage disequilibrium occurring at 1.6 cM on average in the derivatives was comparable to what Maccaferri *et al*.^27^ reported for a worldwide collection of 1,000 spring wheat accessions, indicating a relatively high recombination rate in the DT696 derivatives. Despite the presence of recombination within the 15.8 Mbp of the 5A2 QTL interval inferred from GWAS, further resolution could not be confidently inferred. This may imply the presence of a complex genetic architecture underlying the FHB resistance conferred by the 5A2 QTL with the presence of multiple minor effect genes working together in the interval. Such gene clustering is not unusual as Steiner *et al*.^13^ identified two FHB resistance QTL within the interval of *Qfhs.ifa-5A* using a near-isogenic recombinant inbred line population. These QTL were genetically 0.1 cM apart but associated with 120 Mbp apart intervals on the physical map of chromosome 5A. Research aimed at map-based cloning of *Fhb1* has suggested the presence of at least two resistance genes in the interval, one encoding a chimeric lectin with agglutinin and a pore-forming toxin-like domain and the other a putative histidine-rich calcium-binding protein^17,19^.The identification of at least two sharp declines in the GWAS FDR adjusted *P* value within the 5A2–1 and 5A2–2 intervals lends support to the presence of at least two resistance genes. Increasing the population size and selecting for recombinants in the interval as well as precise phenotyping is needed to improve our understanding of the complex genetic architecture of 5A2 QTL.

Recombination was rare in the 5A2–2 interval (1% of lines) impeding further mapping resolution. In the present study, we genotyped 716 DT696 derivatives in order to select recombinant representatives at the 5A1 and 5A2 QTL interval for phenotyping. This likely allowed us to capture all existing recombination events in the DT696 derivatives. Additional recombinants at the 5A2–2 interval could be identified in future studies by stimulating recombination using biotic and abiotic treatments^28,29^ and application of a high resolution radiation haplotype mapping approach^30^.

SNPs associated with the 5A2 FHB resistance QTL were validated for marker-assisted selection on breeding lines of 13 AAFC SCRDC crosses. Not only did the application to contemporary breeding material allow the validation of SNPs for marker-assisted selection, it also allowed discriminating the effect of *VRN1* from other genes conferring FHB resistance in the interval due to homogeneity at the *VRN1* locus. The breeding populations were subjected to phenotypic selection for lines with reduced levels of FHB by the breeders. Two thirds of selected lines had either Hap 1 (DT696 alleles across the 5A2 interval) or Hap 4 (combination of DT696 and DT707 alleles genotypes in the 5A2 interval), indicating that genotypic selection agreed with phenotypic selection in 75% of cases. Combining genotypic with phenotypic selection could enhance selection gain in the breeding programs^31^ over the 75% obtained using phenotypic selection alone in the present study. We were able to rule out an association of inoculation method with the expression of FHB resistance, by comparing the FHB index of haplotypes at Carman nursery with spray inoculation at 50% anthesis and Brandon with corn spawn inoculation. Because Hap 4 was significantly different from Hap 5 at both nurseries despite different inoculation methods, the inoculation method was not the source of variation. The resistance mediated by Hap 4 is not solely due to developmental traits as spray inoculations at 50% anthesis minimizes the possibility of disease escape associated with plant height and maturity^32,33^.

Forty-six GTWC lines with the DT696 haplotype provide opportunity at the international level for breeding resistance to FHB. Additional research is required to validate that these lines express resistance to FHB in their target environment. As expected, population size raised the recombination frequency at 5A2–2 interval. Assessing the phenotype of germplasm with the recombination events at 5A2–2 interval will be valuable to refine the interval towards identifying candidate genes.

The FHB resistance locus on chromosome 6B in the DT696 derivatives was not detected from the previous QTL mapping study of the DT707 × DT696 population. This suggests that resistance alleles are contributed from founder lines crossed with DT696 and its derivatives. Comparative mapping indicated that the interval defined by SNPs on chromosome 6B in our current study correspond to the Type II FHB resistance QTL detected from *T. turgidum* ssp. *carthlicum* line Blackbird^34^. A pedigree search of DT696 derivatives lines with resistance alleles in the 6B QTL indicated that all were derived from a single breeding population named A0580 (DT696/A0200H-056/3/Langdon-Dic3A (a resistant recombinant substitution line derived from crosses of *T. turgidum* spp. *durum* cv. Langdon with wild emmer wheat *T. turgidum* spp. *dicoccoides*)//SC9475-CX1*2/ND2710 (a hexaploid source of FHB resistance presumably derived from line Sumai 3^35^) and most of them also carried the DT696 haplotype at 5A2 QTL. Considering the field location-dependent expression of both the 5A2 and 6B QTL in the DT696 derivatives, lines of A0580 with resistance alleles of both QTL are valuable source for developing cultivars with consistent expression of resistance over multiple environments.

GWAS analysis of DT696 derivatives could not associate any SNPs within the 5A1 QTL interval with FHB resistance. Consistent detection of the 5A1 QTL in the QTL mapping study using the DT707 × DT696 population in the same field locations used for GWAS of the derivatives (Morden nursery in 2016 and 2017)^2^ minimized the possibility of environment affecting the expression of this QTL. Unlike the 5A2 QTL, the expression of 5A1 seems to be dependent on other loci or certain genetic backgrounds that were rarely present in DT696 derivatives. Weak expression of 5A1 QTL below a level detectable by the GWAS algorithm could be another reason the 5A1 locus was not detected in the derivatives.

More work is needed to further resolve the 5A2 FHB resistance QTL. Efforts are underway to develop Near Isogenic Lines (NILs) carrying recombination break points in the 15.8 Mb interval of the 5A2 FHB resistance QTL. Recombination break points will be mapped with the SNP markers identified in the present study and selected recombinants will be subjected to intense phenotyping to improve the mapping resolution. The NILs will also be subjected to further reverse genetic analysis to identify reliable markers required for high-precision MAS.

In conclusion, phenotyping of DT696 derivatives that carried historical recombination from 72 breeding populations, enabled selecting for early-maturing semi-dwarf lines with FHB resistance equal to or better than DT696 as candidates for developing resistant cultivar. GWAS resolved the 25.8 Mbp 5A2 interval obtained from the bi-parental QTL mapping into a 15.8 Mbp interval. Like *Fhb1* and *Qfhs.ifa-5AS*, a complex genetic architecture is proposed for the 5A2 FHB resistance QTL, with the presence of at least two distinct loci (5A2–1-and 5A2–2) in the interval. GWAS along with haplotype analysis in the contemporary breeding lines enabled the validation of the 5A2 FHB resistance QTL in multiple genetic backgrounds, lending support to the transferability of resistance conferred by this locus to elite germplasm. This study led to the identification of the SNP markers desired for marker-assisted selection that will contribute to the speed and precision of gene pyramiding and should pave the way for improving FHB resistance in durum wheat.

## Materials and Methods

### Plant materials

A search was conducted within pedigrees of AAFC SCRDC durum wheat lines to identify DT696 derivatives among advanced breeding lines using an in-house script of Statistical Analysis System (SAS) software version 9.3 (SAS Institute Inc., Cary, NC, USA) that enabled retrieval of pedigrees from crosses made with DT696 and its secondary and tertiary derivatives. This identified 72 populations with a total number of 716 lines derived from DT696 (Supplementary file 3 online). GWAS was conducted on 223 lines selected based on expressing a moderate FHB resistance phenotype in breeding trials conducted prior to the present study. These lines were also selected to sample recombination at the two FHB resistance QTL on chromosomes 5A (5A1 and 5A2) reported from QTL mapping of the DT707 × DT696 population^2^. Thirteen contemporary breeding populations active in the breeding program were used for marker validation. These populations totalling 523 lines were developed from crosses between advanced breeding lines and the cv. Transcend which is a moderately susceptible durum variety derived from DT696^4^.

### FHB, plant height and relative maturity phenotyping

Phenotyping 223 DT696 derivatives for FHB resistance, plant height and relative maturity was conducted following the method described by Sari *et al*.^2^ at Morden and Brandon locations in 2016 and 2017. The experiments were conducted as an augmented randomized block design with 31 entries per incomplete block including DT696 as resistant and DT707 as susceptible checks. Plots were 1 m long single rows with 50–100 seeds per row. The FHB nurseries were inoculated with corn spawn colonized with a mixture of aggressive 3-acetyl-deoxynivalenol (3ADON) and 15-acetyl-deoxynivalenol (15ADON) producing strains of *F. graminearum*. The Morden nursery was inoculated approximately 2–3 weeks prior to heading, while at the Brandon nursery the first application of corn inoculum was done at six weeks after planting followed by another application two weeks after the first application. Colonized corn grains were broadcasted between the rows at the rate of 40 g m^−2^ at Brandon, and at 8 g per row performed twice, one week apart at Morden. The nursery at Brandon was irrigated three times a week with an overhead low-pressure mist irrigation system immediately upon completion of inoculation to provide moisture for inoculum development in the corn and to provide moisture on the spikes for disease development. The nursery at Morden was irrigated three times a week using Cadman Irrigation Travellers with Briggs booms.

Phenotyping of the 523 contemporary breeding lines was conducted in FHB nurseries located at Brandon and Carman in 2017. Lines at the Brandon nursery were subjected to the same inoculation protocol as DT696 derivatives lines with the experiment conducted as an unreplicated alpha-lattice field designs with 12 entries per incomplete block. At the Carman nursery, the spikes of the plots were spray-inoculated using a CO_2_ pressurized backpack sprayer calibrated at 2 kPa at 50% anthesis with 50 ml per row conidia suspension composed of a mixture of aggressive 3ADON and 15ADON producing strains of *F. graminearum*. The concentration of the conidial suspensions was adjusted to 5 × 10^4^ conidia mL^−1^ using a hemocytometer and Tween 20 (1 drop per 100 ml) was added to the suspension. Inoculation was repeated 4 d after the first inoculation. The nursery was mist irrigated in the evening, and the morning after each inoculation.

FHB incidence (percentage of spikes showing symptoms) and severity (percentage of spike area infected) were recorded for each plot. FHB index was calculated from the incidence and severity rating data using the formula (incidence × severity)/100. Plant height was measured on a representative plant from the soil surface to the tip of spikes excluding the awns. Relative maturity was rated using a 1–4 scale (1 = latest and 4 earliest maturity) concurrent with the FHB rating at three weeks post anthesis, by pinching the seeds and comparing their moisture levels with the parents.

### Genotyping and haplotype profiling of lines

A subset of DT696 derivatives (401 lines) were genotyped using the wheat iSelect 90 K SNP genotyping assay following the method described by Wang *et al*.^23^. The SNP clustering was performed in Genomestudio software v. 2011.1 (Illumina Inc., San Diego, CA, U.S.A.) using the default clustering algorithm and following the workflow described by Cavanagh *et al*.^36^. Approximately 10% of total SNPs (9043 in total) were scorable. The order of markers on the physical map of the IWGSC Refseq v1.0^37^ and durum wheat cv. Svevo^38^ and wild emmer wheat accession Zavitam reference assemblies^39^ was determined by aligning the 90 K SNP source sequences using the Nucmer program available in Mummer v.3 software^40^. Only SNPs with missing data less than 10% and minor allele frequency of higher than 5% were retained. The retained SNPs were then used to calculate an identify-by-state genetic similarity matrix for all possible pairs of lines using Plink v1.07^41^. For lines showing ≥ 0.99 genotypic similarities, only one representative line with the lowest number of missing SNPs was maintained.

To select recombinant representatives at the 5A1 and 5A2 FHB resistance QTL on chromosome 5A, SNPs within the QTL interval were used for genotyping 374 additional DT696 derivatives. SNPs within the 5A1 and 5A2 interval were converted to Kompetitive Allele-Specific PCR (KASP) markers using the Polymarker pipeline^42^. Lines were then genotyped with a Fluidigm (Fluidigm Canada Inc., Ontario, Canada) high-throughput genotyping system using nine KASP markers within the 5A1 and seven KASP markers within the 5A2 QTL interval. The genotypic data of these lines were then combined with those of the 401 lines obtained from the iSelect 90 K SNP genotyping assay and a similarity matrix was generated using Flapjack v. 1.16.10.31 software^43^ to select 359 representative recombinant lines for phenotyping. Lines from the contemporary breeding populations used for validation of SNPs for MAS were genotyped using the same KASP markers with the Fluidigm high-throughput genotyping system.

### GWAS analysis of DT696 derivatives

Of 359 derivatives phenotyped at multiple locations, 223 genotyped using iSelect 90 K SNP genotyping assay, were subjected to GWAS. When markers were filtered for missing data and minor allele frequency (using the criteria described above), 6231 SNPs met the criteria for GWAS. Centered identity-by-state kinship matrix was calculated for these SNPs using Tassel v. 5^44^. The population structure was inferred from the model-based quantitative assessment of sub-population available in Faststructure v1.0 software^45^. Linkage disequilibrium (LD) squared allele frequency correlation (*r*^*2*^) was calculated for each pair of SNPs within each chromosome using Haploview software^46^. The overall pattern of LD decay in the population was estimated following the method described by Maccaferri *et al*.^27^ from pairwise LD *r*^*2*^ estimates of all chromosomes plotted against the genetic distance between SNPs inferred from the tetraploid wheat consensus genetic map^24^. A non-linear regression model was then fitted using the nonlinear least squares method in R v. 3.5.3 software (https://www.r-project.org/). The genetic distance (cM) at which the *r*^*2*^ was lower than the empirical threshold of 3.0 was considered as the average LD decay of the population.

GWAS was conducted with the 6,231 SNPs, FHB traits (incidence, severity and index), plant height and relative maturity data using compressed mixed linear (cMLM)^47^ and Settlement of MLM Under Progressively Exclusive Relationship (SUPER)^48^ models of R package GAPIT v. 2 software^49^ with the kinship matrix generated as described above. The following models were tested with each of cMLM and SUPER methods: 1) no correction for population structure; 2) model corrected for population structure using the % membership coefficient generated by Faststructure for number of subpopulations (Q) of 3–7. The model with correction for the population structure of Q4 was superior to other models according to the quantile-quantile (QQ) plot generated by GAPIT, hence the probability (*P*) values of this model were used for determining the significant SNPs. The experiment-wise *P* value threshold was either 0.05 when the FDR adjusted *P* values were reported or based on Bonferroni adjusted *P* value which was equal to a marker-wise *P* value of 8.25 × 10^−6^. Assigning significant SNPs to QTL was implemented through anchoring the SNPs to the tetraploid wheat consensus genetic map^24^ and durum wheat cv. Svevo genome assembly^38^, and comparing the genetic and physical position of QTL reported in the previous QTL mapping study^2^. The FDR adjusted *P* values were plotted against LD *r*^*2*^ values extracted from Haploview to associate recombination break points with changes in significance of *P* values.

### Haplotype and recombination break points available in the Global Tetraploid Wheat Collection (GTWC)

Haplotypes and recombination breakpoints in the GTWC were based on the genotypic data generated using the 90 K iSelect SNP genotyping assay described by Maccaferri *et al*.^38^. The haplotype and recombination break points were compared between the GTWC and DT696 derivatives at 22 SNPs within the 5A2 QTL interval. Since DT696 and cv. Strongfield were common to both datasets and cv. Strongfield had an identical haplotype to DT707 for the 22 SNPs spanning the 5A2 interval, we could reclassify the genotypic data of the GTWC to either a DT696 or DT707 haplotype. Lines with missing records or heterozygous at these 22 SNPs were excluded from analysis. One-dimensional hierarchical cluster analysis and haplotype visualization was performed using the dendextend and ComplexHeatmap packages of R v.3.5.3.

### Statistical analyses

Variance and covariance analyses were conducted using SAS v.9.3. To estimate the variance due to line, the mixed model was used with genotype assigned as a fixed effect and block assigned as random effect. To estimate the variance due to genotype when plant height and maturity were assigned as covariates, the mixed model was used with line and either plant height or relative maturity assigned as fixed effects, while block was assigned as a random effect. Spearman’s rank correlation analysis was conducted between each of the FHB traits and plant height and maturity using PROC CORR of SAS v.9.3. The significant difference between lines of different haplotypes was inferred using a two-tailed *t*-test with correction for heteroscedastic distribution (two samples with unequal variance) of data in Microsoft Excel.

## Supplementary information

Supplementary Information 1.

Supplementary Information 2.

Supplementary Information 3.

Supplementary Information 4.

## Data Availability

All data generated or analysed during this study are included in this article and its supplementary information files.
