# Supplementary material for: Historic recombination in a durum wheat breeding panel enables high-resolution mapping of Fusarium head blight resistance quantitative trait loci
Source: Sci Rep. 2020 May 5;10:7567. doi: 10.1038/s41598-020-64399-1 (PMC7200731; doi:10.1038/s41598-020-64399-1)
Supplement: Supplementary file 4 — Supplementary Information 4. [file 41598_2020_64399_MOESM4_ESM.pdf]

Historic recombination in a durum wheat breeding panel enables high-resolution mapping of Fusarium head blight resistance quantitative trait loci

Ehsan Sari[1], Ron E. Knox[2*], Yuefeng Ruan[2*], Maria Antonia Henriquez[3], Santosh Kumar[4], Andrew J. Burt[5], Richard D. Cuthbert[2], David J. Konkin[1], Sean Walkowiak[6#], Heather L. Campbell[2], Asheesh K. Singh[7], Jay Ross[2], Prabhath Lokuruge[2], Emma Hsueh[1], Kerry Boyle[1], Christine Sidebottom[1], Janet Condie[1], Shawn Yates[2], Curtis J. Pozniak[6], Pierre R. Fobert[8]

[1]Aquatic and Crop Resource Development Centre, National Research Council, Saskatoon, SK, Canada

[2]Swift Current Research and Development Centre, Agriculture and Agri-Food Canada, Swift Current, SK, Canada

[3]Morden Research and Development Centre, Agriculture and Agri-Food Canada, Morden, MB, Canada

[4]Brandon Research and Development Centre, Agriculture and Agri-Food Canada, Brandon, MB, Canada

[5]Ottawa Research and Development Centre, Agriculture and Agri-Food Canada, Ottawa, ON, Canada

[7]Department of Agronomy, Iowa State University, Ames, Iowa, United States of America

[6]Department of Plant Sciences, University of Saskatchewan, Saskatoon, SK, Canada

[8]Aquatic and Crop Resource Development Centre, National Research Council, Ottawa, ON, Canada

# current address: Department of Plant Sciences, University of Saskatchewan, Saskatoon, SK, Canada

†current address: Canadian Grain Commission, Winnipeg, MB, Canada

Correspondence: *ron.knox@canada.ca; *yuefeng.ruan@canada.ca

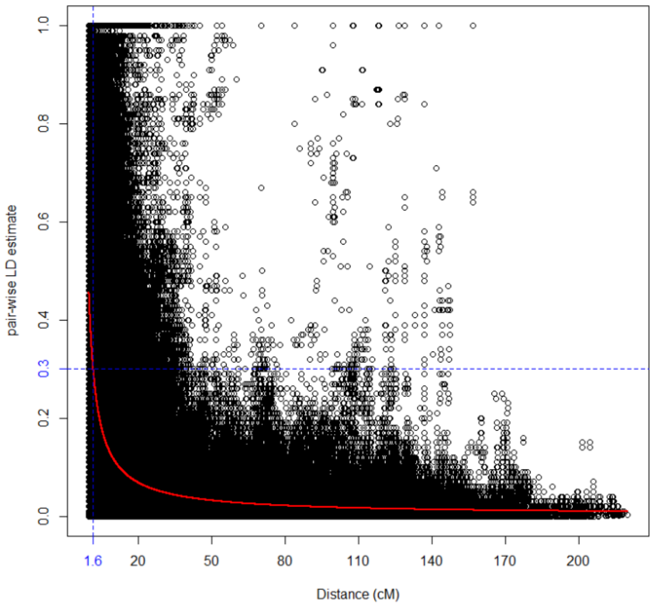

Figure S1. The linkage disequilibrium decay over genetic distance. Pair-wise Single Nucleotide Polymorphism (SNP) LD squared correlation coefficients (r^2) were plotted against the pair-wise genetic distance of markers in durum wheat consensus map of Maccaferri et al. [12]. The red line represents the regression model fitted to the data. The blue dashed line shows the genetic distance interval with the empirical LD r^2 threshold of 0.3.

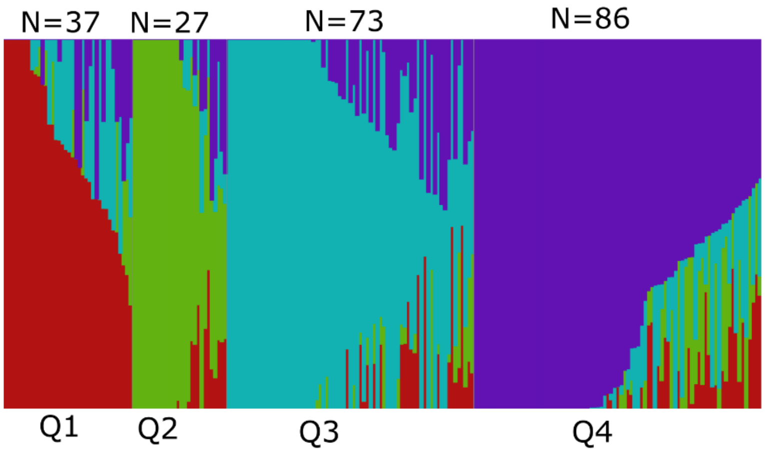

Figure S2. Analysis of population structure and sub-populations (Q) present in DT696 derivatives. Number of lines (N) belonging to each subpopulation is shown on the top of histogram.

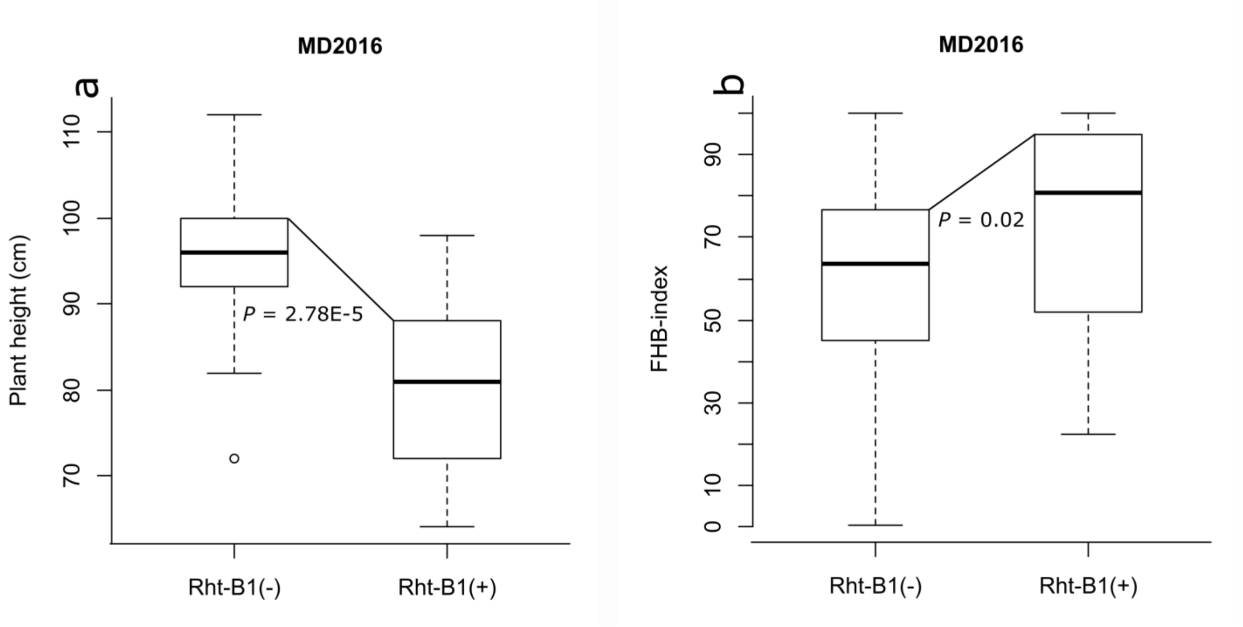

Figure S3. Difference between DT696 derivatives carrying alternate alleles at single nucleotide polymorphism Tdurum_contig27834_260 located within the major wheat plant height gene *Rht-B1* in plant height (a) and Fusarium head blight index (b). *P* value denotes the significance of difference between lines with (+) and without (-) the semi-dwarfing allele in plant height and FHB index. Plant height and FHB index are from the Morden (MD) field trial in 2016, where Tdurum_contig27834_260 was significantly associated with plant height.

Table S1. Analysis of variance (F-test) probability (*P*) value of line mean of Fusarium head blight, plant height, and relative maturity traits of the field trials conducted for 223 DT696 derivative lines and covariance analysis of plant height and plant maturity with FHB traits. The field trials were conducted as an augmented randomized block design with one replicate at each of Morden and Brandon nursery in 2016 and 2017. *P* value of line mean was estimated by a mixed model when line was assigned as a fixed effect and block as random effects. For covariance analysis, the *P* value of the line mean was estimated by a mixed model when line and covariate (either plant height or maturity) were assigned as fixed effects and block was assigned as random effects.

Location-year	Traits	Variance analysis *P* value	Covariance analysis (covariate plant height) *P* value	Covariance analysis (covariate plant maturity) *P* value
Morden-2016	FHB incidence	0.9	0.6	0.9
	FHB severity	0.7	0.9	0.12
	FHB index	0.8	0.3	0.1
	Plant height	0.3	-	-
	Plant maturity	0.6	-	-
Morden-2017	FHB incidence	0.07	0.1	0.1
	FHB severity	0.0005	0.002	0.0002
	FHB index	0.0004	0.0017	0.0003
	Plant height	0.0008	-	-
	Plant maturity	0.3	-	-
Brandon-2016	FHB incidence	0.5	0.6	0.6
	FHB severity	0.0013	0.0035	0.0015
	FHB index	<0.0001	<0.0001	<0.0001
	Plant height	<0.0001	-	-
	Plant maturity	0.4	-	-
Brandon-2017	FHB incidence	0.07	0.1	0.1
	FHB severity	0.0003	0.0010	0.0011
	FHB index	<0.0001	<0.0001	<0.0001
	Plant height	<0.0001	-	-

Plant maturity	0.3	-	-

Table S2. Spearman's rank correlation between FHB traits (incidence, severity and index) and plant height and maturity of the field trials conducted for 223 DT696 derivatives. Significance levels are denoted by '*' for *P* < 0.05, and '**' for *P* < 0.01**.**

Location-year	FHB trait	Plant height	Plant maturity
Morden-2016	incidence	-0.10*	0.16**
	severity	-0.02	0.13*
	index	-0.05	0.16**
Morden-2017	incidence	-0.23**	0.28**
	severity	-0.28**	0.23**
	index	-0.29**	0.28**
Brandon-2016	incidence	-0.31**	0.18**
	severity	-0.44**	0.20**
	index	-0.44**	0.21**
Brandon-2017	incidence	-0.41**	0.25**
	severity	-0.39**	0.21**
	index	-0.44**	0.24**
